# Reaction–Diffusion Patterning of DNA-Based Artificial Cells

**DOI:** 10.1021/jacs.2c06140

**Published:** 2022-09-14

**Authors:** Adrian Leathers, Michal Walczak, Ryan A. Brady, Assala Al Samad, Jurij Kotar, Michael J. Booth, Pietro Cicuta, Lorenzo Di Michele

**Affiliations:** † Biological and Soft Systems, Cavendish Laboratory, University of Cambridge, Cambridge CB3 0HE, U.K.; ‡ Department of Chemistry, Faculty of Natural and Mathematical Sciences, King’s College London, London SE1 1DB, U.K.; ¶ Chemistry Research Laboratory, University of Oxford, Oxford OX1 3TA, U.K.; § Department of Chemistry, University College London, London WC1H 0AJ, U.K.; ∥ Department of Chemistry, Imperial College London, Molecular Sciences Research Hub, London W12 0BZ, U.K.; ⊥ fabriCELL, Imperial College London, Molecular Sciences Research Hub, London W12 0BZ, U.K.

## Abstract

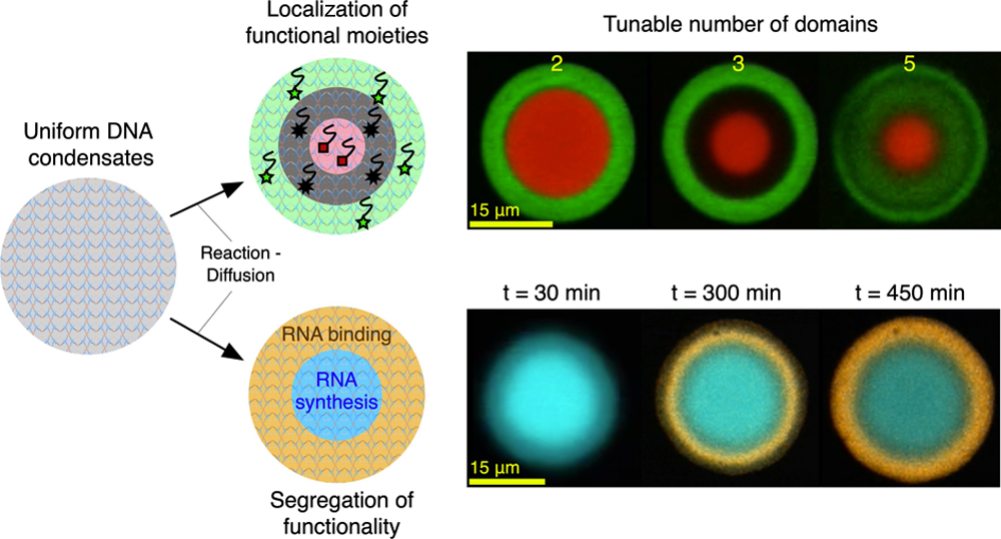

Biological cells display complex internal architectures with distinct micro environments that establish the chemical heterogeneity needed to sustain cellular functions. The continued efforts to create advanced cell mimics, namely, artificial cells, demands strategies for constructing similarly heterogeneous structures with localized functionalities. Here, we introduce a platform for constructing membraneless artificial cells from the self-assembly of synthetic DNA nanostructures in which internal domains can be established thanks to prescribed reaction–diffusion waves. The method, rationalized through numerical modeling, enables the formation of up to five distinct concentric environments in which functional moieties can be localized. As a proof-of-concept, we apply this platform to build DNA-based artificial cells in which a prototypical nucleus synthesizes fluorescent RNA aptamers that then accumulate in a surrounding storage shell, thus demonstrating the spatial segregation of functionalities reminiscent of that observed in biological cells.

## Introduction

Bottom-up synthetic biology aims to engineer artificial systems that exhibit biomimetic structures and functionalities via the rational combination of molecular and nanoscale elements. These systems often take the form of artificial cells (ACs), microrobots constructed *de novo* to replicate a subset of the behaviors typically associated with biological cellular life, including communication, adaptation, energy conversion, and motility.^1−3^ Despite still being far from the complexity of live cells, ACs are regarded as promising technological platforms for personalized healthcare, where cell-like microdevices could operate *in vivo*, detect disease-related biomarkers, and respond by synthesizing and releasing therapeutic agents, potentially resulting in minimally toxic and efficient treatments.^1,4−7^ Similarly, ACs could underpin innovations in synthesis, through the optimized production of materials and pharmaceuticals and in environmental remediation by selectively capturing and storing pollutants.^1,4,5,8,9^

ACs often rely on microcompartments constructed from lipid,^10,11^ polymer,^11,12^ or protein membranes,^13^ but membraneless implementations based on coacervates^14−19^ or hydrogels^18−21^ are gaining traction, which is driven by their enhanced robustness and easy manufacture as well as the renewed interest in biomolecular condensates and membraneless compartments in cell biology.^22,23^ Similar to the case of biological condensates, the accumulation of target molecules within membraneless ACs can be induced through selective affinity for the scaffold phase without relying on a membrane.

**Figure 1:**
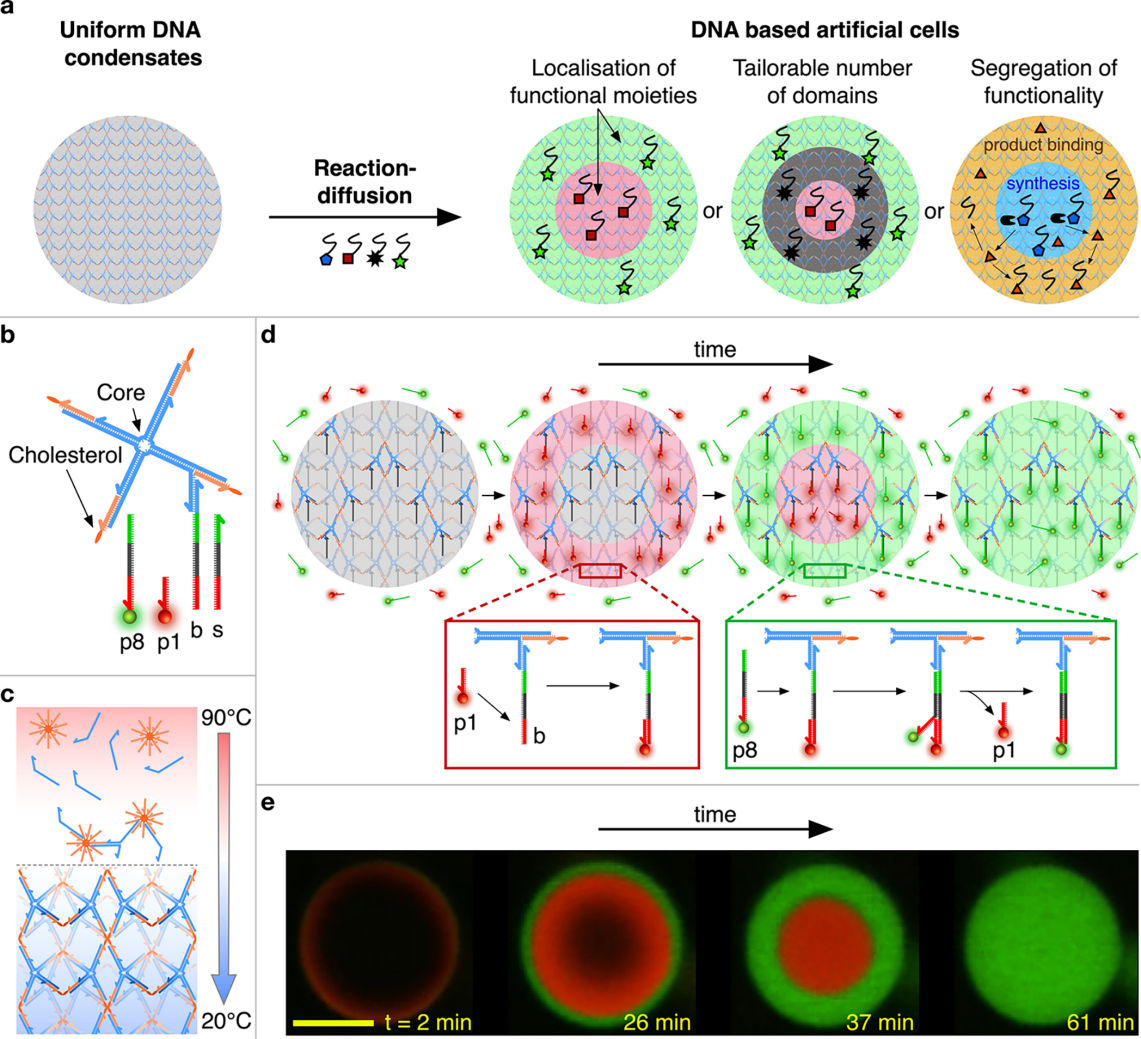
Reaction–diffusion patterning of amphiphilic DNA condensates. (a) Reaction–diffusion processes are used to pattern initially uniform DNA condensates and construct DNA-based artificial cells featuring distinct internal environments of controllable number and molecular makeup, unlocking the spatial engineering of functionality. (b) Foundational building block of the condensates, consisting of a locked four-way DNA junction with cholesterol moieties at the end of each arm. The constructs are composed of four distinct strands that form the junction (blue) and four identical cholesterolized oligonucleotides (orange).^31−33^ One arm features an additional overhang connected to a base strand (b), which serves as a binding site for complementary freely diffusing patterning strands. The latter range between 16  (p1) and 40 nt (p8) in length and compete over (color-coded) overlapping binding domains on the base strand. p1 and p8 shown here are functionalized with Alexa 594 (red) and Alexa 488 (green) fluorophores, respectively. The stop strand (s) has the same sequence as the base strand and can be added in solution to sequester the excess patterning strands. Sequences of all the DNA oligonucleotides are provided in Table S1. (c) Assembly process for amphiphilic DNA condensates. Samples containing all single-stranded DNA components are slowly annealed from 90 to 20 °C, leading to the formation of a nanoporous framework in which the branched DNA motifs connect micelle-like hydrophobic cores where the cholesterol modifications localize, as previously reported.^31−33^ Sample preparation details are provided in the Experimental Methods (SI). (d) Schematic depiction of the designed reaction–diffusion pathway. At time *t* = 0, condensates are exposed to a solution of p1 (short, red) and p8 (long, green) patterning strands in excess concentrations compared to the base strands. Short p1 DNA strands are able to diffuse inside the condensates faster than long p8 strands, allowing for prior binding to the base strand (red box). At later times, p8 strands then diffuse within the condensates and, due to the sequence design, are able to displace p1 strands via toeholding^36,37^ (green box). The result is a sequence of two fronts that propagate inward through the condensate. (e) Series of confocal micrographs of the process discussed in panel c, where propagating fronts are visualized thanks to fluorescent modifications of p1 and p8. the scale bar represents 15 μm.

Like (eukaryotic) cells, AC implementations can benefit from a heterogeneous internal architecture that regulates the transport and spatial distribution of (bio)molecules, which can facilitate the design of biomimetic pathways that require the colocalization or separation of specific compounds.^24^ However, while with membrane-based platforms it is relatively easy to establish internal heterogeneity, for instance, through nesting or sequential assembly,^24^ no general platform has been proposed to program the local composition in membraneless scaffolds. Here we leverage the structural and dynamic programmability afforded by DNA nanotechnology^25,26^ to construct membraneless condensates of DNA nanostructures, which can be “patterned” thanks to a reaction–diffusion scheme^27−30^ (Figure 1a). This strategy can generate up to five chemically addressable, distinct microenvironments in a concentric geometry, whose features can be rationalized through numerical modeling. As a proof-of-concept, we use the platform to create model ACs with a spatially resolved functionality, namely, where a fluorescent RNA aptamer is synthesized in a prototypical “nucleus” and accumulates in an outer shell (Figure 1a, right).

## Results and Discussion

Our condensates self-assemble from branched amphiphilic DNA nanostructures, as shown in Figure 1b. Similar constructs were previously demonstrated to form nanoporus phases with programmable structures, molecular-sieving properties, stimuli responsiveness, and the ability to host dynamic DNA circuitry.^31−35^ As depicted in Figures 1c and S1 and detailed in the Experimental Methods (SI), spherical aggregates with cell-like dimensions (10–40 μm in diameter) readily emerge from a one-pot annealing reaction. Small-angle X-ray scattering measurements demonstrate that the condensates display internal crystalline order with a BCC unit cell and lattice parameter of 26.8 nm, which is consistent with previous observations on similar systems (Figure S2).^32^

The foundational building block used here consists of a DNA four-way junction, where the end of each 35 base-pair (bp) duplex arm is label by cholesterol moieties (Figure 1b). One of the arms was modified with an additional single-stranded overhang to which a base (b) strand was connected. The base strand serves as a competitive binding site for freely diffusing patterning strands (p) of different lengths, as depicted in Figure 1b, where the complementarity of domains is shown by the same coloration and opposite directionality. All the patterning strands feature an identical (red) domain complementary to the base, but for longer strands the complementarity is extended to more adjacent domains. This feature allows any longer patterning strand to displace a shorter strand from the base *via* toehold-mediated strand displacement (toeholding)^36,37^ but not *vice versa*, establishing a length-dependent binding hierarchy. The length of even the shortest binding domain (14 nucleotides (nt)) is such that the thermal detachment of the patterning strands does not occur within experimentally relevant time scales. Sequences of all oligonucleotides are reported in Table S1.

The principle for AC patterning is schematically depicted in Figure 1d. Condensates are prepared hosting uniformly distributed base strands but without any initially connected patterning strands. Multiple types of patterning strands are then introduced in solution in excess concentrations compared to the number of available binding sites in the condensates (see Experimental Methods (SI)). In this example, we introduce patterning strands p1 (16 nt) and p8 (40 nt) labeled with an Alexa 594 (red) and Alexa 488 (green), respectively. Note that we label patterning strands with numbers that increase with the strand length such that p(*n* + 1) is longer than p*n*. The diffusion coefficient of DNA decreases with the contour length.^38^ This dependency is enhanced in porous environments like our condensates,^39^ allowing shorter DNA strands to diffuse significantly faster than longer ones. The shorter patterning strands, p1 in the example, will thus rapidly access the condensate, occupying base strands progressively from the outside of the condensate inward. At later times, the longer p8 strands also diffuse through the condensate and, as they do so, displace p1 strands from the base strands via toeholding and release them back into solution. The result, experimentally demonstrated through confocal micrographs in Figure 1e, is a sequence of inward-propagating fluorescent fronts: a red wave corresponding to the rapidly diffusing p1 appears first, which is then replaced by a green front produced by the slowly diffusing but strongly binding p8 strands. Note that in confocal measurements the signal from excess patterning strands in solution is not visible due to the comparatively much higher concentration of binding sites within the condensates.

This scheme thus allows us to localize different oligonucleotides in distinct and individually addressable concentric shells within the condensates. While in this example the patterning strands bear a simple fluorescent modification, one could easily envisage the inclusion of functional elements, thus paving the way to establishing a spatially resolved functionality in membraneless ACs.

**Figure 2:**
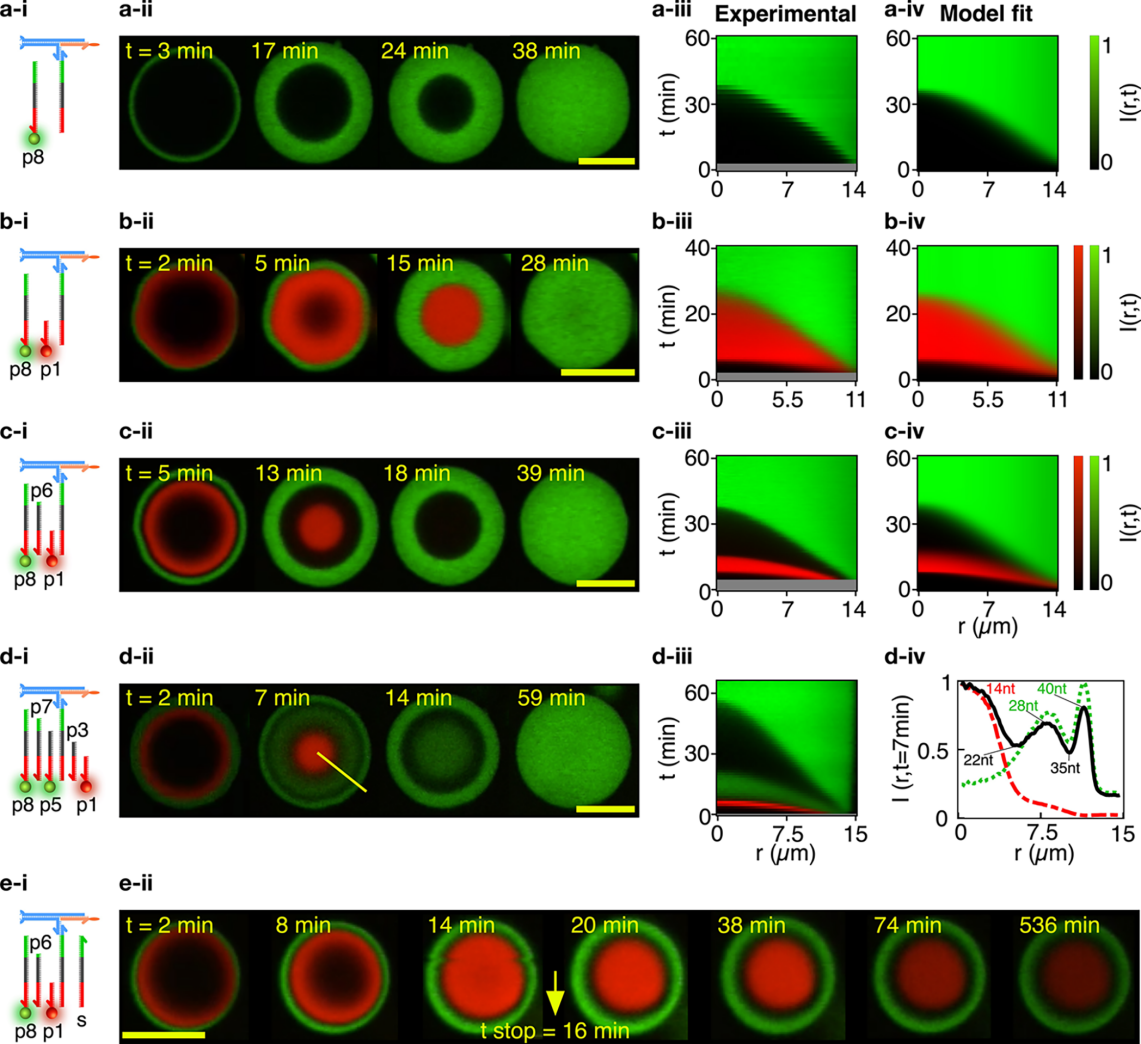
Condensate patterning is predictable and customizable. (a–d) Patterning-strand scheme (-i) and equatorial confocal microscopy sections (-ii) for condensates patterned to form an increasing number of concentric domains, from one in system a to five in system d. Some patterning strands are fluorescently labeled with Alexa 594 (p1) and Alexa 488 (p5 and p8) while others do not bear modifications, resulting in dark regions intermitting the fluorescent shells in the confocal data. See Table S1 for the DNA sequences. The spatiotemporal evolution of the domain structure is visualized as the azimuthally averaged, normalized radial intensity profile *I*(*r*, *t*), where *r* is the radial coordinate defined from the centroid of the condensate and *t* is the time elapsed from exposure of the condensates to the patterning strands (-iii). For systems a–c, *I*(*r*, *t*) is compared with the fitted outcome of a reaction–diffusion numerical model (**-iv**). Note that early times are not shown in experimental color maps (gray bands) due to a delay between the time at which condensates were exposed to the patterning strand (*t* = 0) and the start of the confocal recording. See the Experimental Methods (SI) for information on image analysis and numerical modeling. For system d, subpanel d-iv shows the radial intensity profiles extracted from confocal images at *t* = 7 min, highlighting the presence of five distinct domains. The green dotted and red dashed lines mark the signals from the Alexa 488 (p5 and p8) and Alexa 594 (p1) channels, respectively, while the black solid line represents the overall intensity. All profiles are normalized by their highest value. (e) Domain propagation can be arrested by adding an excess of the stop strand (s) in solution (e-i, see also Figure 1a), as demonstrated in e-ii with confocal data for a system with three patterning strands (p1, p6, and p8). The stop strand was added at *t* = 16 min, after which no further pattern evolution was observed (besides photobleaching). Videos S1–S8 show the pattern evolution in individual condensates (even numbered) and larger fields of view (odd numbered). See the supplementary videos key in the SI. Scale bars represent 15 μm.

Domain structure and evolution can be programmed via the number and length of patterning strands, as summarized in Figure 2. Confocal micrographs for representative condensates exposed to one, two, three, and five patterning strands are shown in Figure 2a–d-ii, while Videos S1–S12 exemplify pattern evolution in individual condensates and larger sample areas (see the supplementary videos key in the SI). In Figure 2a–d-iii, the spatiotemporal evolution of the patterns is captured by 2D color maps showing the (azimuthally averaged and normalized) radial profile of the fluorescence intensity, *I*(*r*, *t*), where *r* is the distance from the centroid of the condensate and *t* is the time elapsed from exposure to the patterning strands (see Experimental Methods (SI)). Examples of analogous color maps for multiple condensates and different numbers of patterning strands are shown in Figures S3–6. For tests with more than two domains, nonfluorescent (dark) patterning strands of lengths intermediate to the two fluorescent ones were used. For instance, as shown in Figure 2c, a dark 30 nt strand (p6) is used in combination with 16 nt (red) p1 and 40 nt (green) p8, generating a dark shell that separated the fast-propagating red wave and the slow-propagating green wave. Two dark (p3 and p7) and three fluorescent strands (p1, p5, and p8) are used in Figure 2d, generating five distinct microenvironments at ∼7 min from exposure to the patterning strands, as highlighted by the azimuthally averaged fluorescent intensity profiles (Figure 2d-iv). Note that in this case the difference in length between adjacent species varies between 5 and 7 nt, demonstrating a separation ability comparable to those of electrophoretic techniques and hinting at the possible applications of ACs in the detection and separation of nucleic acids. For a fixed number of patterning strands, the relative widths of the domains can be controlled by the design, as shown in Figure S7 for three-domain experiments in which dark strands of different lengths were used.

In all examples discussed, the reactions are designed to progress toward the equilibrium configuration in which the longest, most stable construct occupies all binding sites, defying the purpose of our strategy as a means of engineering an internal AC architecture. However, pattern evolution can be readily arrested using a stop strand (s, Figure 1a), with a sequence identical to that of the base. The stop strand is added in solution in an excess concentration compared to that of all patterning strands combined (see Experimental Methods (SI)), sequestering them and interrupting wavefront propagation. Figure 2e-ii shows that patterns arrested with this protocol remained stable for several hours, as required for the purpose of spatial engineering in ACs. The lack of any visible blurring of the patterns confirms the absence of internal diffusion of the amphiphilic DNA building blocks that make up the structure of the condensates.

**Figure 3:**
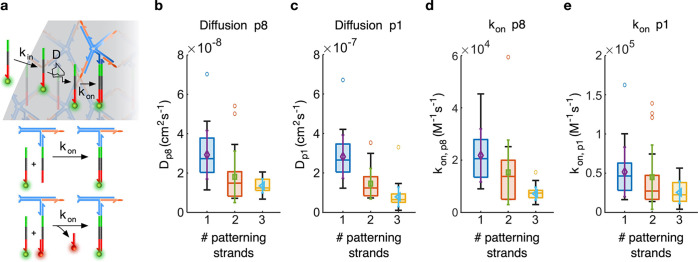
Model fitting enables the extraction of reaction–diffusion parameters. (a) Schematic representation of the color parameters, which consist of an entry rate *k*_in_, a diffusion constant *D*, and a binding or displacement rate *k*_on_ (top). With *k*_on_, we indicate both the second-order binding rate of a patterning strand to a free binding site and that of the toehold-mediated strand displacement process through which a longer patterning strand replaces a shorter one that previously occupied a binding site (bottom, see the Modeling Methods (SI). (b and c) Diffusion coefficients for the 40 nt patterning strand p8 and the 16 nt patterning strand p1, respectively. (d and e) Binding rates for the 40 nt patterning strand p8 and the 16 nt patterning strand p1, respectively. Data are shown for samples with one patterning strand (p1 or p8; Figures 2a, S3, and S4; *N* = 33 condensates for p1 and *N* = 29 condensates for p8), two patterning strands (p1 and p8; Figures 2b and S5; *N* = 23 condensates) and three patterning strands (p1, p6, and p8; Figures 2c and S6; *N* = 43 condensates). The results are displayed as box plots with highlighted median, upper, and lower quartiles (box); 50th centile (whiskers) outliers are excluded. Overlaid on the box plots are the means (symbol) and standard deviations (error bar the same color as the symbol) of the distributions.

The system’s evolution can be modeled through a set of coupled reaction–diffusion equations under the assumptions of a spherical condensate geometry and an excess of patterning strands in solution, as fully detailed in the Modeling Methods (SI). Alongside known or easily determined system parameters such as condensate size, the model requires as inpust the diffusion constants (*D*) of the patterning strands, the second-order rate constants through which the patterning strands bind the base or displace previously bound strands (*k*_on_), and the exchange rate of patterning strands between the bulk and the condensate (*k*_in_).^40,41^ The latter three quantities (*D*, *k*_on_, and *k*_in_), which are graphically depicted in Figure 3a, are used as fitting parameters. The model also features a partition coefficient of the patterning strands within the condensates;^40^ for realistic values, this coefficient was found to have no significant effect on the fitting outcomes and was thus set to 1 (Figure S8).

The model outputs the spatiotemporal evolution of the concentration of bound patterning strands within the condensates, which, after accounting for diffraction-induced blurring and normalization, renders an estimate of the experimental fluorescent intensities. This model can thus be used to fit the experimental *I*(*r*, *t*) data for up to three competing patterning strands (see Modeling Methods (SI)). Qualitatively comparing the experimental and fitted *I*(*r*, *t*) maps demonstrates their good agreement, as can be seen in Figures 2a–-c-iv and S3–S6. A quantitative assessment of the fit residuals, shown in Figure S9, confirms the good match, with deviations typically within ±10–15% for data associated with long (p8) patterning strands and within ±20–25% for short (p1) strands. The larger deviation observed for the shorter patterning strands can be ascribed to the smaller data sets given that short strands experience a shorter reaction–diffusion transient. In Figure S10 we further show histograms of the residuals that combine values from all sampled condensates. The distributions appear quite symmetrical but deviate from a normal profile. A non-normal distribution may result from the small shape differences between the modeled and experimental reaction–diffusion profiles, which is also highlighted in the residual maps (Figure S9). These differences may emerge due to early time effects related to sample mixing, optical artifacts due to refractive index mismatches and absorption, nonspherical condensate geometry, or nonisotropic diffusion caused by contact with the bottom of the experimental chamber.

Panels b and c of Figure 3 show the distributions of fitted diffusion constants and binding coefficients, respectively, for our shortest (p1, 16 nt) and longest (p8, 40 nt) patterning strands, as determined for experiments with a single patterning strand (p1 or p8; Figures 2a, S3, and S4), two patterning strands (p1 and p8; Figures 2b and S5), and three patterning strands (p1, p6, and p8; Figures 2c and S6).

We observe an order-of-magnitude difference in the diffusion constant between p1 and p8, with *D*_p1_ = 1–4 × 10^–7^  cm^2^ s^–1^ and *D*_p8_ = 1–4 × 10^–8^  cm^2^ s^–1^. *D* is the primary parameter that determines the propagation speed of the reaction–diffusion fronts through the condensates; therefore, the difference found between *D*_p1_ and *D*_p8_ is consistent with expectations. We further note that both diffusion constants decrease in the presence of additional patterning strands, hinting at crowding effects.

Panels d and e in Figure 3 show the fitting outcomes for the rate constant *k*_on_. In all cases, given that p1 is unable to displace other incumbent strands, *k*_on,p1_ describes the binding of p1 to a free base strand. Similarly, *k*_on,p8_ describes hybridization to free base strands in experiments only featuring p8. When shorter patterning strands are present alongside p8, *k*_on,p8_ can be interpreted as the effective second-order rate constant of the toehold-mediated strand displacement reaction through which p8 displaces incumbent p1 (two-strand case) and p6 (three-strand case).^36^ See Figure 3a (bottom) and Modeling Methods (SI) for a quantitative justification of this interpretation. Fits produce values of *k*_on,p1_ and *k*_on,p8_ within the same order of magnitude (10^4^–10^5^ M^–1^ s^–1^), with the former slightly larger than the latter. Values are consistent with previous observations for hybridization in hydrogels^28^ and smaller than rates typically measured in freely diffusing constructs (∼10^6^ M^–1^ s^–1^).^36^ The similarity between the rates of hybridization (*k*_on,p1_) and toeholding (*k*_on,p8_) is expected given that the two are known to converge for toehold lengths in excess of 6 nt, a condition that is verified in our reactions.^36^ A decreasing trend was found for an increasing number of patterning strands, which was more pronounced for *k*_on,p8_ compared to *k*_on,p1_ and may thus be due to crowding.

The material exchange rate *k*_in_, as summarized in Figure S11, is slightly larger for p1 compared with p8, which is consistent with the higher diffusion rate of the shorter patterning strand. Meanwhile, no significant trend was observed as a function of the number of patterning strands.

To explore possible correlations between the fitting parameters and to gauge the robustness of our fits, we computed maps of the sum of squared residuals, χ^2^, as a function of each pair of parameters (Figure S12).^42−44^ The maps reveal a degree of correlation between *D* and *k*_in_, while *k*_on_ is not correlated to either variable. All maps, however, show a clear minimum, hinting at the identifiability of all parameters, which was confirmed by the individual likelihood curves and associated identifiability analysis (Figure S13). We note that while *k*_on_ is identifiable, the model displays a relatively weak sensitivity to this parameter for values in excess of ∼10^5^ M^–1^ s^–1^ (Figures S12a and S13a). This weakened sensitivity can be rationalized as the result of the diffraction blurring applied in the model, which for sufficiently large values of *k*_on_ dominates over the blurring of the propagating front induced by finite reaction rates (see Modeling Methods (SI) for details).

**Figure 4:**
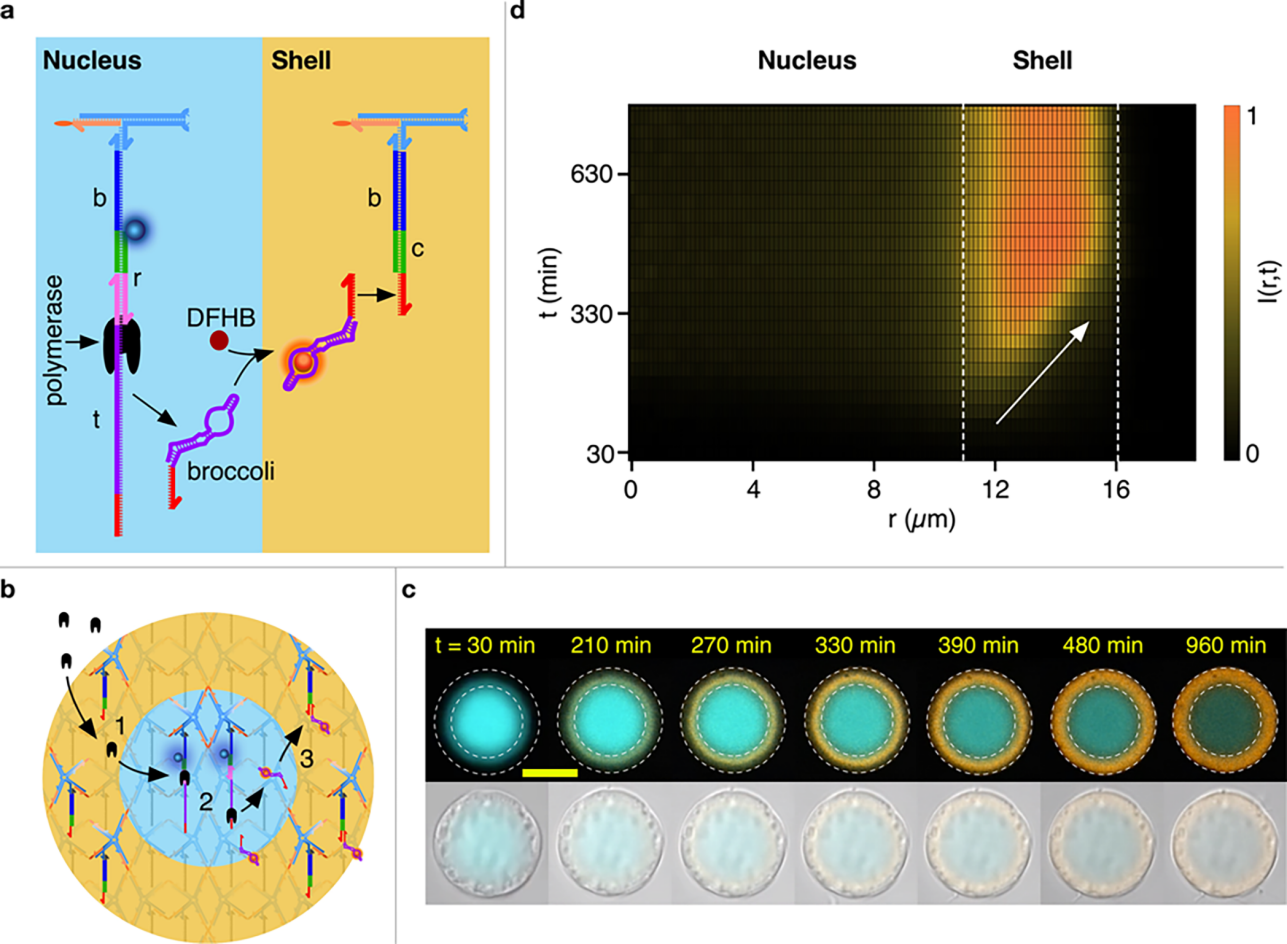
Spatially distributed functionality in a model artificial cell. (a) Schematics of the functional nucleic acid machinery in the nucleus (cyan) and shell regions (orange). In the nucleus, connected to the base strand are a bridge (r) strand and the template (t) strand. Together, these form a double-stranded T7 promoter (pink) and a single-stranded polymerase template (purple, red) from which a polymerase (black) is able to synthesize Broccoli RNA aptamers (folded purple and red). These aptamers then form a complex with DFHBI molecules to become fluorescent (orange). The base strands in the shell region are connected to capture strands (c) with single-stranded overhangs (red) complementary to a free domain on the broccoli aptamer. Complementary DNA and RNA domains are shown in the same color. Protocols for patterning the ACs are detailed in the Experimental Methods (SI). (b) Mode of operation of the AC. The polymerase is added in solution alongside NTPs, DFHBI, and other components required for Broccoli synthesis, which diffuse through the shell (1) to reach the nucleus, where the aptamers are produced (2). The aptamers then diffuse outward and bind to the dedicated sites in the shell (3). (c) A series of confocal images (top) of an AC progressively building the Broccoli aptamer in the shell (orange). Note how the signal accumulates from the nucleus–shell interface and propagates outward. The nucleus is shown in cyan and progressively photobleaches. The dashed lines mark the physical boundary of the AC and that between the nucleus and the shell. The bottom images of bright-field images of the same AC overlaid onto (faint) confocal data, demonstrating that no physical change to the AC occurs during Broccoli synthesis. The reaction is initiated at time *t* = 0, as discussed in the Experimental Methods (SI). (d) Color map showing the evolution of the radial fluorescent intensity of the aptamer. The slope of the fluorescent front signals accumulation from the inside out, as highlighted by the white arrow. Dashed lines mark the nucleus–shell and shell/–olution boundaries. Videos S13–S16 show the responses of multiple ACs with different shell–nucleus size ratios. The scale bar represents 15 μm.

Having identified a route for establishing addressable domains in condensates, we can proceed with localizing functional elements within different regions to create a model AC that hosts a spatially organized pathway. As summarized in Figure 4a, the AC was patterned with a “nucleus” region hosting a template construct (t), which contains a T7 RNA polymerase promoter sequence and the DNA sequence complementary to an RNA aptamer, and the shell region containing binding sites for the RNA product. The template is anchored to the base strand through a bridging (r) strand, which ensures the formation of the double-stranded T7 promoter required for the T7 polymerase to begin transcription.^45^ The RNA produced was a modified version of the DFHBI-binding fluorescent Broccoli aptamer.^46^ Addition of an extra 8 bps to the stem region produced a significantly brighter aptamer, as discussed in the Experimental Methods (SI) and Figure S14. The aptamer also features a binding site complementary to the single-stranded overhang present in the capture (c) strands located in the shell region. Note that because the r–t complex located in the nucleus is longer than the capture strand present in the shell, the patterning of these devices needed to be conducted following a multistep protocol, as detailed in the Experimental Methods (SI) and Figure S15. The protocol also involves washing steps to remove any unbound r–t complexes that would result in RNA synthesis occurring in the bulk solution.

The sought response at the AC level is sketched in Figure 4b. Patterned ACs are exposed to a polymerase that, similar to other proteins of comparable size,^32^ can diffuse through the condensates, reaching the template in the nucleus. Here, the Broccoli aptamer, which readily binds to DFHBI, is produced, diffuses out toward the shell, and binds the capture motifs. These constructs would thus display the envisaged separation of functionality in addition to a basic form of communication between two domains, one producing a signal in the form of RNA constructs and the other receiving it.

Figure 4c (top) shows a time-resolved sequence of confocal micrographs from an AC that produced the designed response. The nucleus (cyan), fluorescently stained thanks to a fluorophore on the bridging strand, remained the same size through the experiment and only underwent progressive bleaching. In turn, fluorescence from the Broccoli aptamer (orange) builds up in the shell region from the inside out, consistent with the RNA product being produced in the nucleus. Combined bright-field and confocal micrographs of the same objects confirm that the overall size and appearance of the condensate does not change during Broccoli accumulation (Figure 4c, bottom). A color map of the radial intensity of the Broccoli emission versus time is shown in Figure 4d, where the outward-propagating front, marked by an arrow, is clearly observable. Data from more ACs with different shell thicknesses relative to nucleus size are summarized in Figure S16, while time lapses of the process can be inspected in Videos S13–S16. As a control, in Figure S17 we show the results of experiments with template or promoter constructs free in solution, which as expected show outside-in accumulation. Finally, in Figures S18 and S19 we report the time-dependent Broccoli emission in bulk fluorimetry experiments, including samples with aptamer-expressing ACs and samples containing only the supernatant solution but no ACs. The lack of signal from the latter further confirms that aptamer production occurs exclusively within the AC nucleus.

## Conclusion

In summary, we have introduced a general process for the creation of stable and individually addressable domains in condensates self-assembled from DNA nanostructures. The process relies on a reaction–diffusion scheme, where patterning constructs with different diffusivities and binding affinities compete for binding sites within the condensates. The number and size of the domains can be tuned by design and predicted by numerical modeling. As a proof-of-concept, we adapted the patterning scheme to construct a model DNA-based artificial cell with an active nucleus that produced a fluorescent RNA aptamer and a storage shell where the product progressively accumulated. With this basic implementation as a starting point, one could envision future developments toward artificial cells capable of producing, storing, and later releasing therapeutic RNA elements, such as small-interfering RNAs.^47^ These implementations, however, would need to also encapsulate the RNA polymerase and NTPs rather than relying on freely diffusing enzymes and building blocks, which could be achieved by surrounding the artificial cell with a less permeable barrier.

More generally, we argue that reaction–diffusion-patterned DNA condensates could constitute a versatile platform for engineering cell-like agents with spatially- and temporally resolved functionalities. Potential responses are not limited to the expression or capture of RNA products, as the domains can be enriched with virtually any functional molecule or nanoscale agent that can be linked to the patterning strands, including enzymes, nanoparticles, aptamers, and photoresponsive elements, thus unlocking the opportunity to engineer evermore complex reaction pathways within the ACs. Finally, while here DNA condensates are used as passive scaffolds, one can envisage design modifications where patterning alters the local physical structure. For instance, one could ensure that the localization of responsive moieties makes specific regions in the condensates sensitive to targeted degradation (photoinduced or enzymatic), enabling the creation of voids within the artificial cells that could be used to store bulky cargoes or simply regulate the connectivity of the remaining domains. In turn, dynamic changes in the local mesh size could enable the design of pathways that couple changes in local transport properties with biochemical activity, giving rise to more complex time-dependent responses. For example, one could envisage a negative feedback loop where the local accumulation of an RNA product represses transcription by (sterically) hindering polymerase diffusion and configurational freedom. If coupled with enzymatic RNA degradation, similar systems could sustain fluctuations in RNA expression periodic in time and space, reminiscent of the oscillating gene expression in biological cells.^48^ Similarly, RNA expression could be coupled to changes in the artificial cell size, leading to mechanical actuation useful for engineering propulsion or shape changes in artificial tissues.

## Supporting Information Available

All experimental, image analysis, numerical modeling and fitting methods; bright-field microscopy images; SAXS characterizations; I(r, t) patterning maps; data demonstrating control over domain thickness; analysis of the fitting model behavior; correlation between fitting parameters and their identifiability; performance of the extended Broccoli aptamer; patterning protocol for the RNA-synthesizing ACs; RNA synthesis in ACs; control experiments; nuclotide sequences; and supplementary video descriptions (PDF)
Large view of one patterning strand (p8) (AVI)
Zoomed-in view of one patterning strand (p8) (AVI)
Large view of two patterning strands (p1 and p8)(AVI)
Zoomed-in view of two patterning strands (p1 and p8)(AVI)
Large view of three patterning strands (p1, p6, and p8) (AVI)
Zoomed-in view of three patterning strands (p1, p6, and p8) (AVI)
Large view of five patterning strands (p1, p3, p5, p7 and p8) (AVI)
Zoomed-in view of five patterning strands (p1, p3, p5, p7 and p8)(AVI)
Large view of three patterning strands (p1, p6, and p8) and the stop strand (AVI)
Zoomed-in view of three patterning strands (p1, p6, and p8) and the stop strand (AVI)
Large view of one patterning strand (p1) (AVI)
Zoomed-in view of one patterning strand (p1) (AVI)
Broccoli RNA production and storage in artificial cells (example 1) (AVI)
Broccoli RNA production and storage in artificial cells (example 2) (AVI)
Zoomed-in view of Broccoli RNA production and storage in artificial cells (fluorescence) (AVI)
Zoomed-in view of Broccoli RNA production and storage in artificial cells (bright-field and light fluorescence) (AVI)

## Supplementary Material

ja2c06140_si_001.pdf

ja2c06140_si_002.avi

ja2c06140_si_003.avi

ja2c06140_si_004.avi

ja2c06140_si_005.avi

ja2c06140_si_006.avi

ja2c06140_si_007.avi

ja2c06140_si_008.avi

ja2c06140_si_009.avi

ja2c06140_si_010.avi

ja2c06140_si_011.avi

ja2c06140_si_012.avi

ja2c06140_si_013.avi

ja2c06140_si_014.avi

ja2c06140_si_015.avi

ja2c06140_si_016.avi

ja2c06140_si_017.avi

## Notes

A dataset associated to this publication is available free of charge at https://doi.org/10.17863/CAM.87545.
